# Comparative Assessment of Push-Out Bond Strength and Dentinal Tubule Penetration of Different Calcium-Silicate-Based Endodontic Sealers

**DOI:** 10.3390/dj12120397

**Published:** 2024-12-06

**Authors:** Mihai Merfea, Sanda Ileana Cimpean, Radu Stefan Chiorean, Aurora Antoniac, Ada Gabriela Delean, Iulia Clara Badea, Mindra Eugenia Badea

**Affiliations:** 1 Faculty of Dentistry, Iuliu Hatieganu University of Medicine and Pharmacy, 400012 Cluj-Napoca, Romania; mihai.merfea@umfcluj.ro (M.M.); ada.delean@umfcluj.ro (A.G.D.); iulia.badea@umfcluj.ro (I.C.B.); mebadea@umfcluj.ro (M.E.B.); 2 Faculty of Automotive, Mechatronics and Mechanical Engineering, Technical University of Cluj-Napoca, 400114 Cluj-Napoca, Romania; radu.chiorean@rezi.utcluj.ro; 3 Faculty of Material Science and Engineering, National University of Science and Technology Politehnica Bucharest, 060042 Bucharest, Romania; aurora.antoniac@upb.ro

**Keywords:** endodontic sealer, bioceramic sealer, calcium silicate sealer, push-out, SEM

## Abstract

**Background:** Adhesion within endodontic obturation material and root canal walls improves the efficacy of the endodontic treatment by establishing a barrier that inhibits reinfection and entombs residual bacteria. This study evaluates the push-out bond strength (POBS) of calcium silicate sealers compared to an epoxy-resin-based sealer. **Methods:** A total of 36 extracted mono-radicular teeth were prepared with Pro Taper Ultimate and irrigated with 5.25% sodium hypochlorite and 17% EDTA. The specimens were randomly split into three groups (*n* = 12) according to the endodontic sealer and filling technique used as follows: Ah Plus with the continuous wave condensation technique (CWC), Ah Bioceramic (Ah Bio) with the single-cone technique, and Total Fill Hi-Flow (FKG Hi-Flow) with the CWC technique. The material was allowed to set for 4 weeks, and afterwards, the roots were placed in acrylic resin and sectioned into 1 mm transverse slices. A POBS test was conducted using a universal testing machine, and the mode of bond failure was assessed at 4× magnification using a stereomicroscope. Six specimens from each group were selected for SEM-EDX examination to evaluate dentinal tubule penetration. The data were analysed using analysis of variance and Tukey and Bonferroni post hoc tests. **Results:** The POBS tests revealed higher values for Ah Plus in comparison to both calcium silicate sealers (*p* < 0.001), while FKG Hi-Flow showed superior results to Ah Bio (*p* < 0.001). The cohesive mode of failure was prevalent in all three groups. **Conclusions:** In conclusion, the resin-based sealer showed higher bond strength and better dentinal tubule penetration than the two calcium silicate sealers tested, while FKG Hi-Flow outperformed AH Bio.

## 1. Introduction

The main goal of endodontic treatment is to thoroughly disinfect and fill the root canal system. This objective is driven by the need to prevent or treat apical periodontitis. Because of the impossibility of obtaining a sterile environment in a complex endodontic space, the filling of the root canal is essential for the treatment outcome [1]. The purpose is to achieve an adequate bond between the dentinal walls and the root canal filling material. The endodontic filling material must block the development of residual bacteria and interrupt any leakage between the endodontic space and the periapical tissues [2].

Filling a root canal is commonly achieved by using a solid core, mainly gutta-percha, and a sealer [3]. The sealer must cement and bond the core to the dentinal walls. The gutta-percha can be used either in the “warm” or “cold” condensation technique, while the sealer helps to avoid any void formation and eventually seals lateral or accessory canals [4]. An optimal endodontic sealer offers good sealing, dimensional stability, biocompatibility, insolubility in tissue fluids, adhesion with dentinal walls, and plentiful setting time to ensure enough working time [5].

The commercially accessible sealers are divided, based on the material criterion, into the following categories: glass ionomers, resins, zinc–oxide–eugenol, silicones, calcium hydroxide, and bioceramics [6]. Bioceramic-based endodontic sealers are composed of calcium silicate and/or calcium phosphate and have engaged much attention regarding their physicochemical and biological properties [7].

Calcium-silicate-based sealers were first developed for cold-filling techniques [8]. AH Plus Bioceramic (Ah Bio) (Dentsply, Konstanz, Germany) is a new injectable sealer, containing zirconium dioxide (50–70%) as a radio opacifier component, along with tricalcium silicate (10–15%) as the bioactive constituent [9]. Ah Bio is recommended to be used in a cold-filling technique by the manufacturer, mainly, the single-cone technique (SC) [10]. Total Fill BC Sealer (FKG Dentaire, La Chaux-de-Fonds, Switzerland) and BioRoot RCS (Septodont, Saint Maur des Fosses, France) were proven to have an increase in viscosity following heat application [11]. To address these disadvantages and to support the use of calcium-silicate-based sealers in combination with the continuous wave condensation technique (CWC), Total Fill BC Sealer Hi-Flow (FKG Hi-Flow) (FKG Dentaire, La Chaux-de-Fonds, Switzerland) was developed [12]. It is, to our knowledge, the only sealer specifically designed to accomplish all the requirements for a sealer to be used with warm vertical root filling techniques. A study by Casino-Alegre et al. revealed that FKG Hi-Flow exhibits better dentinal tubule penetration used with heat application techniques rather than cold-filling techniques [13]. Before, epoxy-resin- and zinc–oxide–eugenol-based sealers were largely accepted to be stable and indicated in warm root-filling techniques [14].

AH Plus (Dentsply, Konstanz, Germany) is an epoxy-resin-based sealer consisting of two paste–paste components [15]. Thanks to its remarkable characteristics, like low solubility, bonding to dentin, and excellent sealing ability, it is considered the benchmark in endodontic sealers, and it is commonly used in clinical practice [16]. However, it has no antibacterial action, and it shrinks when setting [17].

No interdependence between leakage and adhesion to dentin was demonstrated [18]. Calcium-silicate-based sealers showed better penetration of the dentinal tubules [16], lower dye leakage [19], and higher dislodgement resistance [20] in previous studies.

Assessment of the adhesion strength of root filling materials to the dentinal walls was carried out using the push-out bond strength test (POBS) [21]. This test applies a compressive load perpendicular to the dentinal tubules, simulating clinical stress in prepared root canals with a natural canal anatomy [22], developing a real-life load case comprising mainly shear stress, in contrast to usual tensile bond tests [23]. The failure modes can be either when the filling material breaks its adhesion to the dentinal wall, resulting in an adhesive failure, or a cohesive mode of failure when the material fractures internally [24]. However, no research has been conducted to our knowledge that evaluates the bond strength and dentinal tubule penetration of a calcium-silicate-based sealer developed specifically for warm-filling techniques compared to bioceramic sealers developed for cold techniques. Therefore, our study designed three experimental groups based on the recommended technique: FKG Hi-Flow with CWC, Ah Bio with SC, and Ah Plus with CWC as the control group.

The main objective of this study was to analyze the bond strength between the root canal wall and three different endodontic sealers (Ah Plus, FKG Hi-Flow, Ah Bio) used with the specific filling technique recommended by the manufacturer for each material (CWC or SC). The secondary goal was to assess the failure mode of these sealers and the correlation between dentinal tubule penetration and bond strength. The null hypotheses were that there is no difference between the bioceramic and the epoxy-resin-based sealers concerning bond strength and that there is no disparity between these materials regarding the mode of failure and tubule penetration.

## 2. Materials and Methods

### 2.1. Tooth Selection

Thirty-six monoradicular human teeth, freshly extracted for periodontal purposes were chosen for this study. The Ethics Committee of the University of Medicine and Pharmacy “Iuliu Hatieganu” Cluj-Napoca approved this study on 16 May 2023, approval number AVZ86, and all the patients signed informed consent for using their extracted teeth. All the roots were observed under a stereomicroscope (Leica M320, Leica Microsystems GmbH, Wetzlar, Germany) at 4× and 20× magnification to rule out any caries, cracks, or root resorptions. Buccal and proximal radiographs (Planmeca, Helsinki, Finland) were used to exclude any teeth without a single root canal and a single apical foramen and to exclude oval root canals or any defect, like caries and root resorptions, obstacles, or previous root canal treatments. Oval root canals were defined as having a ratio between buccal–lingual and mesial–distal diameters greater than 2, when measured on the buccal and proximal radiographs at 9 mm from the radiological apex [25]. The Schneider criteria [26] were used to calculate the root canal curvature and exclude root canals with more than 5° curvatures [27].

### 2.2. Sample Preparation

After disinfection with sodium hypochlorite and tissue debris removal, the teeth were kept in a saline solution before preparation. Round diamond burs (Edenta, St. Gallen, Switzerland) were used for access cavity preparation. All the crowns were cut with a diamond disc (Microdont, Sao Paulo, Brazil) to obtain a standardized working length of 17 mm [27]. The working length was calculated by subtracting 1 mm from the measurement between the apical foramen and the coronal landmark of the first instrument used (ISO 10 K-File (VDW, Munich, Germany)) [16]. The presence of the K-file at the apical foramen was verified using the stereomicroscope. All the root canals were prepared with the ProTaper Ultimate (Dentsply, Konstanz, Germany) sequence up to FX (size 35) using an X-Smart Plus (Dentsply, Konstanz, Germany) electric-driven motor following the manufacturer’s settings and procedure. Between each file, the root canal was irrigated with 2 mL NaOCl 5.25% (Cerkamed, Stalowa Wola, Poland) using a side-vented irrigation needle (Cerkamed, Stalowa Wola, Poland). The final irrigation consisted of 3 mL EDTA 17% (Cerkamed, Stalowa Wola, Poland), followed by 3 mL NaOCl 5.25%, with a flushing speed of 1 mL per minute [28].

Afterward, the specimens were randomly allocated into three groups (*n* = 12) and obturated using three different sealers and the technique indicated by the manufacturer for each material:

Group 1: Ah Plus (Dentsply, Konstanz, Germany, lot no. 2403000500) using the continuous wave condensation (CWC) technique (AH Plus);

Group 2: Total Fill BC Sealer Hi-Flow (FKG Dentaire, Le Cret-du-Locle, Switzerland, Lot no. 2301SPWF) using the CWC technique (FKG Hi-Flow);

Group 3: Ah Plus Bioceramic Sealer (Dentsply, Konstanz, Germany, lot no. KS230221) using the single-cone (SC) technique (AH Bio).

The dedicated master cone Fx (Dentsply, Konsantz, Germany) was examined for tug back at WL, and the root canals were dried using paper cones. All the sealers were mixed using the manufacturer’s instructions and applied in the root canal as follows: for groups AH Plus and FKG Hi-Flow, the sealer was coated homogeneously on the master cone and applied in the root canal, while for the AH Bio group, the sealer was delivered with a lentulo spiral (VDW, Munich, Germany) and coated master cone [29]. Next, the master cones were cut using a heated plugger at the root canal emergence, and the corresponding filling technique was used for each group. All the filling procedures were conducted by a single operator possessing over 5 years of endodontic experience. The access cavities were sealed with the composite, and radiographs were taken to determine the obturation’s quality. The specimens were kept for 30 days in an incubator at 100% humidity in a saline solution and 37 °C temperature [30]. At the end of this period, the specimens were fixed into acrylic resin (Duracryl, Spofa Dental, Jičín, Czech Republic) in a vertical position [31]. Using a water-cooled diamond disc (Ø125 × 0.35 × 12.7 mm Isomet, Buehler, Lake Bluff, IL, USA) running on an Isomet machine (Buehler, Lake Bluff, IL, USA), 1 mm slices were obtained by sectioning the teeth horizontally from the apical part to the cementoenamel junction [24]. Six samples for each root resulted following the sectioning. The samples were classified into apical, middle, and coronal sections [24]. The apical part of every slice was marked to be easily recognizable. All the slices were measured with a digital micrometer (Yato, Wrocław, Poland) with a precision of up to 2 µm, and their exact thicknesses were registered. Both the apical and coronal sides of each slice were analyzed under an Olympus CKX 41 microscope (Olympus, Tokyo, Japan) at 10× magnification to exclude slices with any voids or irregular shapes. Three roots that presented an oval-shaped canal or isthmuses were excluded and replaced. Two perpendicular diameters were measured on each side using Quick Photo MICRO 3.0 software (Promicra, Prague, Czech Republic).

### 2.3. Mechanical Testing

All the root sections were coded and subjected to a compressive load utilizing a universal testing machine (Instron model 3366, Instron Corp., Norwood, MA, USA), at a crosshead speed of 1 mm/min until bond failure occurred [27]. Stainless-steel pluggers of varying diameters between 0.3 mm to 0.9 mm were used to apply the force in an apical–coronal direction. The plugger was selected to cover as close to 85% of the root canal diameter, without touching the walls [32]. The highest load applied on filling upon adhesion loss was registered in Newtons for each specimen and it was used to compute the shear stress value (in MPa). Comparing the sealers based on the unitary load is more appropriate since the value of the force varies with the geometry of the adhesion area. The following formula was used:

τ(MPa)=F/Af

where F denotes maximum force, and Af signifies the adhesion area. Af is the lateral area of a truncated cone considering the thickness of the specimen and the difference in the apical and coronal diameters caused by the taper of the root canal [24].

$$Af(mm^{2})=\frac{Da+Dc}{2}*\pi *g*\frac{1}{10^{6}}$$

where Da represents apical diameter, Dc represents coronal diameter, π = 3.14, and g is the specimen’s thickness. The Da was calculated on the apical side of each slice using Quick Photo MICRO 3.0 software (Promicra, Prague, Czech Republic) by obtaining the average between two perpendicular diameters. The Dc was calculated in the same way, on the coronal side of the slice.

### 2.4. Failure Evaluation

For failure mode determination, each slice was carefully examined by a blinded operator at 4× magnification using a stereomicroscope (Leica M320, Leica Microsystems GmbH, Wetzlar, Germany). The Stelzer classification [33] was used to evaluate the failure mode: adhesive, when the coverage of root canal dentin by sealer was observed to be less than 25%, cohesive when a minimum of 75% of the dentinal surface was covered by filling material, or mixed between 25% and 75% [25].

### 2.5. Dentinal Tubule Penetration Evaluation by Scanning Electron Microscopy (SEM)

Six random specimens from the middle third of the root were selected from each group and examined by a scanning electron microscope (Inspect F50m FEI, Hillsboro, OR, USA) operated at 30 kV. The instrument was equipped with a field emission gun with 1.2 nm resolution and an energy-dispersive X-ray spectrometer (EDX) with 133 eV resolution for the MnK line. All the samples were placed on adhesive carbon tape and coated with Au before analysis. To assess sealer penetrability into the dentinal tubes, each slice was analyzed at 40× magnification to evaluate the sealer–dentin interface. Next, every slice was observed at 4000–5000× magnification in each quarter, and the deepest penetration zone was measured for every specimen.

### 2.6. Statistical Analysis

One-way analysis of variance (ANOVA) for independent samples was employed to evaluate discrepancies between cohorts characterized by endodontic sealers. Pairwise comparisons between the groups were examined utilizing Tukey and Bonferroni post hoc tests. The threshold of statistical significance was established at *p* < 0.05. All the visual representations and analyses were executed employing JASP software (JASP Team 2024, JASP version 0.19.0).

## 3. Results

### 3.1. Results of Mechanical Testing

The mean POBS values for the three groups are shown in Table 1. Pairwise comparison between the groups (ANOVA, Tukey, and Bonferroni post hoc tests) showed significantly higher push-out bond strength for AH Plus than both the calcium-silicate-based materials (*p* < 0.001). Among the bioceramic sealers, FKG Hi-Flow revealed higher dislodgement resistance (*p* < 0.001).

**Figure 1:**
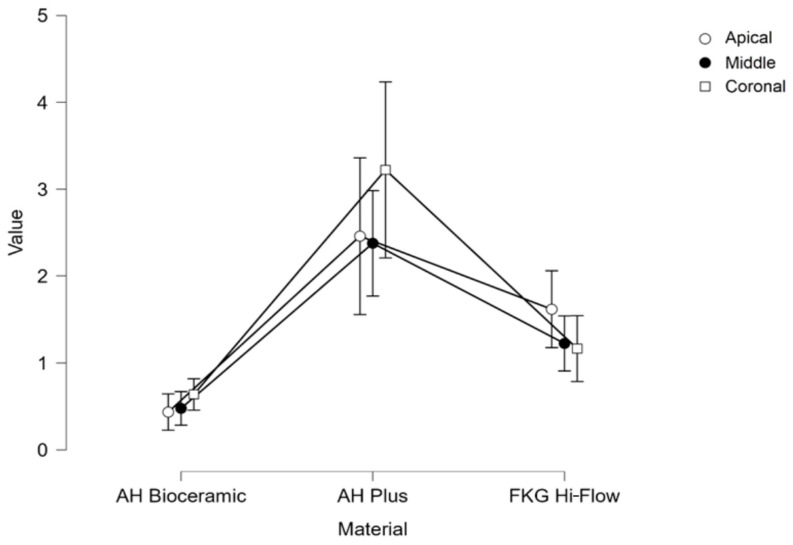
Mean push-out bond strength values (MPa) for each third and each material tested.

When looking at different thirds of the root canal (Table 2), no significant differences were observed between sections in the same sealer group for all three materials individually (*p* > 0.05). AH Plus showed significantly better results (*p* < 0.001) in the apical, middle, and coronal thirds compared to AH Bio and better results than FKG Hi-Flow in the median (*p* < 0.05) and coronal (*p* < 0.001) thirds. The other results were not statistically significant, although they followed the same pattern as the global mean POBS values when sections from the same thirds (apical, middle, and coronal) were compared between groups (Figure 1).

### 3.2. Failure Mode

**Figure 2:**
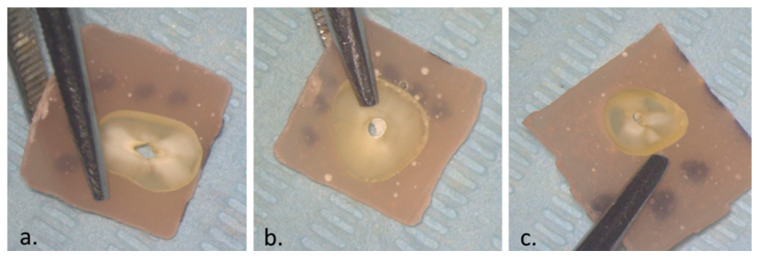
Failure mode analysis by stereomicroscope at 4×: (**a**) adhesive failure; (**b**) cohesive failure; (**c**) mixed failure.

The failure mode analysis results are shown in Table 3 and are exemplified in Figure 2. Ah Bio and FKG Hi-Flow showed a predominantly cohesive failure, while Ah Plus revealed a more equally distributed failure mode but still with the biggest percentage of cohesive failures.

### 3.3. Dentinal Tubule Penetration Results Analyzed by SEM

**Figure 3:**
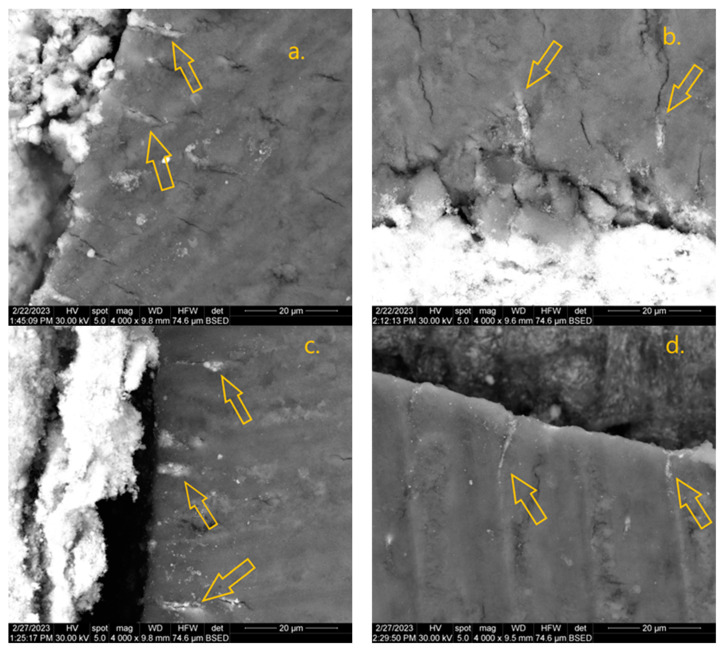

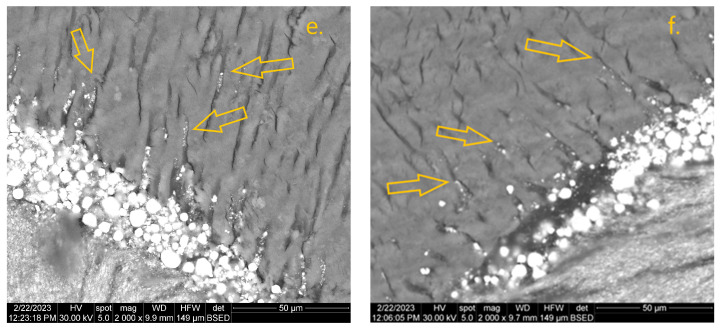
SEM-EDX images. Sealer dentinal tubule penetration is indicated by yellow arrows in slices from the middle third of the root. Sealer appears white. FKG Hi-Flow—(**a**,**b**) (4000× magnification), Ah Bio—(**c**,**d**) (4000× magnification), Ah Plus—(**e**,**f**) (2000× magnification).

The results of the scanning electron microscopy (SEM) dentinal tubule penetration can be seen in Table 4. Ah Plus exhibited the deepest dentinal tubule penetration (with values between 35.3 and 86 µm), followed by FKG Hi-Flow (14 to 30.6 µm) and Ah Bio (7.7 to 19.3 µm). Examples of tubule penetration can be seen in Figure 3. When correlating dentinal tubule penetration with POBS values on the selected slices, a positive correlation was found for Ah Plus and Ah Bio, but without statistical significance, and a negative correlation for FKG Hi-Flow. The results can be seen in Table 4.

## 4. Discussion

The purpose of root canal treatment extends beyond eliminating bacteria by disinfecting and instrumenting the root canal and aims to hermetically seal the endodontic system by creating a single block amid the dentin and the obturation components. Because of the nonexistent adhesiveness of the gutta-percha, the sealing material has the role of developing a chemical link between the dentinal wall and the obturation core. The primary purpose of this research was to analyze the impact of the chemical compositions of three different endodontic sealers on the POBS. Although all three materials allegedly accomplish chemical bonding to the canal wall, significant statistical differences in adhesion strength were found among the tested materials, therefore the null hypothesis was rejected.

Push-out tests have commonly been used to investigate the dislodgement resistance of root canal obturation [34], evaluating the adhesion of the sealing material to the dentinal wall or the gutta-percha [32]. The compressive load test more accurately simulates the clinical conditions, and it is more efficient in measuring bond strength than tensile tests that apply a pull-out force [35,36]. The accuracy of this test can be altered by factors such as instrumentation (diameter and taper), filling technique (cold versus warm technique), test pin diameter, tooth type, slice thickness, load velocity, and core material stiffness [25,27,37]. The use of a 0.35 tip size file with a 12% taper ensured a reproducible preparation of the root canal [27]. Regarding the obturation technique, two studies [38,39] concluded that cold lateral compaction and CWC may affect the dislodgement resistance, although Koki et al. concluded that heat-generating techniques do not influence the POBS on bioceramic sealers [40]. Donnermeyer et al. [14] stated that some calcium silicate sealers may be affected by heat application during warm obturation procedures if the sealer is not specifically developed for this technique. Despite a possible confounding variable because of the use of different techniques on different materials, the current research was formulated to provide information about the POBS of bioceramic sealers in a clinically relevant context. Hence, the materials were employed in accordance with the specifications and guidelines established by the manufacturer, as well as the practices commonly adopted by clinicians.

The most important step for a correct evaluation of the dislodgement resistance seems to be the proportional relationship between the diameter of the puncher and that of the root canal filling [23,32]. A ratio smaller than 55% was found to affect the POBS test in both studies, while a punch size between 70% and 90% did not influence the POBS [23]. Chen et al. stated that a ratio bigger than 85% may impact the POBS test, while it should be sufficiently large in order to avoid its puncturing into the filler material [32]. In our research, punchers of variable sizes were used to be within this range in order to match the filling diameter and were centered on the root canal filling.

Regarding the specimen thickness, Chen et al. [32] concluded it must be 0.6 times greater than the obturation diameter to prevent alterations of the POBS test. In our study, the specimens were 1 mm thick, and the filling diameter ranged from 0.42 mm to 1.25 mm.

The findings of this study show that the POBS of AH Plus were significantly higher than the other sealers, in accordance with the present data [27,41,42,43]. One study concluded the contrary [44]. The contrasting results may be caused by different experimental setups, like the choice of the plugger’s diameter. FKG Hi-Flow showed superior values compared to AH Bio, which do not corroborate with the current data [45]. However, the difference between the studies can be caused by different obturation techniques. Khoury et al. used single-cone filling in both groups, compared to our study, which used the manufacturer’s designated technique for each group. The duration and the type of immersion solution were also reported to affect both resin-based sealers and calcium-silicate-based sealers in reducing the values of the POBS test [46]. Khoury et al. kept the samples for 2 weeks at 100% humidity in an incubator, while in our study, the specimens were kept for 4 weeks at 100% humidity in a saline solution. Therefore, this can be a reason for different POBS values.

Considering the current evidence, it can be assumed that AH Plus has a higher POBS. This can be a consequence of the covalent link between the epoxy resin and the amino groups of root canal walls [47,48], producing a stronger bond in contrast to the interaction between calcium silicates and dentinal tissue. The interaction between the bioceramic sealer’s main components, like calcium silicate and hydroxyapatite, and the radicular dentin produces a “mineral infiltration zone”, a consequence of a process known as alkaline etching [49,50], that may have a weaker connection to the dentinal wall in comparison to epoxy resins. This can be explained by the volume loss during setting [51] and the existence of more gaps at the sealer–dentin interface [52].

When the coronal, medial, and apical thirds were compared for each tested material, no statistically significant difference was found. However, when comparing one material to another, at the level of the same third, AH Plus had significantly better push-out bond strength for each third than AH Bio. FKG Hi-Flow showed significantly lower values in POBS than AH Plus in the coronal thirds. All the other comparisons within the coronal, medial, and apical thirds for the materials tested were not statistically significant.

The secondary objective of this research was to evaluate the failure patterns of the tested materials. Concerning the failure types, FKG Hi-Flow had mostly a cohesive mode of failure, in contrast with another study that revealed a majority of mixed failures [44]. In our study, AH Bio showed a predominantly cohesive failure, while Dewi et al. concluded that this sealer has a mixed failure [44], but other research that used premixed syringe calcium silicate sealers showed mostly cohesive failures [27]. AH Plus returned mixed and cohesive failures, findings that are in accordance with previous studies [27,44]. When considering that both calcium silicate sealers had predominantly cohesive modes of failure, it is likely that the lower POBS values are correlated with cohesive failure. Moreover, it seems that the adhesion of the gutta-percha to the sealer is less powerful compared to the link between the sealer and the dentin. Using a bioceramic-coated gutta-percha cone might create a chemical bond between it and the calcium silicate material and, therefore, improve this [53]. However, Eltair et al. assessed the interfacial connection between Total Fill BC and a coated gutta-percha cone and revealed the presence of a gap between the sealer and the gutta-percha [54]. Regarding the clinical implications of the failure mode, an endodontic sealer should create a tight seal and have good adhesion to both dentinal walls and an obturation core, like gutta-percha [55], while the sealing ability does influence the treatment outcome [56]. A cohesive mode of failure suggests that the bond between the root canal wall and the sealer is more powerful than the bond between the sealer and gutta-percha, while an adhesive failure hints at a weak bond to the dentin [57]. This means that the bacterial apical leakage may appear at different interfaces corresponding to the sealer used [58], resulting in the calcium silicate sealers tested in this study being more vulnerable at the sealer–core interface because of their predominant cohesive failure.

The degree of dentinal tubule penetration increases the contact between root canal walls and obturation materials, therefore promoting good tridimensional filling of the endodontic space. The depth and percentage of the sealer’s penetration are key indicators of its effectiveness, with higher penetration associated with better clinical outcomes [59]. Theoretically, when the sealer enters into dentinal tubules, it creates a lock that should enhance the mechanical adhesion to dentin [60]. Our study reports a non-significant positive correlation for Ah Plus and Ah Bio, in accordance with several studies that showed no significant correlation between dentinal tubule penetration and the POBS test [42,61,62,63]. Verma et al. reported that BIO-C Ion+, a premixed bioceramic sealer, showed better penetration and lower POBS values when compared to AH Plus [41]. A study conducted by Shieh et al. compared Ah Plus and two injectable calcium silicate sealers, Endosequence BC and Ah Bio, and showed no significant correlation between sealer penetration and bond strength for the three sealers or for different thirds of the root canal [44].

Scanning electron microscopy (SEM) has been proven as an alternative to staining sealers with rhodamine B and the use of confocal laser scanning microscopy [31,64]; therefore, in our study, SEM-EDX was used for determining the depth of dentinal tubule penetration. The results are in accordance with other studies, finding the depth of tubule penetration to be relatively small [41,65,66]. Examinations of sections from the FKG Hi-Flow group resulted in lower tubule penetrability than AH Plus in warm vertical compaction but showed better penetrability than AH Bio. This can be explained by the use of a warm filling technique within the FKG Hi-Flow group, which exhibits pressure on the gutta-percha sealer, resulting in better penetrability than cold techniques [63,67]. A study that compared tubule penetration between Ah Plus and Ah Bio using the SC technique showed a higher percentage of tubule penetrability for Ah Bio across the whole root but also in the middle third of the root canal [44]. Our study used sections from the middle third, but the use of CWC for Ah Plus may have exhibited better tubule penetration due to the pressure applied during the filling. Several calcium silicate sealers available in premixed syringes were researched, and they appear to have better or the same penetration depth as epoxy-resin-based sealers [41,42,45,66], in contrast to our study. A reason for this may be the different way of delivering the sealer in the root canal and variations in irrigation protocols, as well as alterations in the sealer’s flowability [68].

The main limitation of this study is that it was conducted in an experimental setup. The setting of calcium-silicate-based sealers can be longer in an incubator with 100% humidity than in a natural environment, where dentinal tubules may contain fluid [69]. Anatomical variations of the extracted teeth may also affect the results of this study, while the laboratory setup of the POBS test may be different than the actual clinical situation. Irrigation protocols could also be a factor influencing the adhesion to the dentinal wall, along with different filling techniques.

## 5. Conclusions

Within the limitations of this study (in vitro study, extracted teeth, controlled setting of the root canal sealers), the following can be concluded:

- Resin-based endodontic sealers had a higher bond strength to the root canal wall than calcium silicate sealers;
- Within the bioceramic sealers tested, FKG Hi-Flow showed better dislodgement resistance than Ah Bio;
- All the tested sealers showed a predominantly cohesive mode of failure;
- A positive correlation between dentinal tubule penetration and bond strength was found for Ah Bio and Ah Plus.

While resin-based sealers remain the benchmark in root canal filling, the use of calcium silicate sealers still requires further investigations to evaluate the adhesion capability of bioceramic sealers in a natural environment; to find ways to improve the sealer–core interface and, thus, apical leakage; and to assess how factors like the root canal filling technique or different irrigation protocols can influence the dentinal tubule penetrability and, ultimately, the clinical outcome.

## Figures and Tables

**Table 1:** Descriptive statistics for push-out bond strength of all sealers tested in MPa.

Material	*n*	Mean	SD	SE	CV
AH Bio	54	0.52	0.39	0.05	0.75
AH Plus	54	2.68	1.68	0.23	0.63
FKG Hi-Flow	54	1.29	0.75	0.1	0.58

**Table 2:** Descriptive statistics for POBS results of each material by root canal thirds (in MPa).

Material	Apical		Middle		Coronal	
	Mean	SD	Mean	SD	Mean	SD
AH Bio	0.43	0.36	0.47	0.42	0.63	0.37
Ah Plus	2.45	1.62	2.37	1.33	3.22	2.03
FKG Hi-Flow	1.61	0.73	1.22	0.71	1.16	0.78

**Table 3:** Mode of failure for each material during dislodgement (in %).

Material	Adhesive	Mixed	Cohesive
Ah Plus	25.0	36.5	38.5
FKG Hi Flow	31.4	25.0	43.6
Ah Bio	13.0	13.0	74.0

**Table 4:** Descriptive statistics of dentinal tubule penetration for each tested sealer (in µm) and push-out bond strength (in MPa) test for the 6 slices analyzed per group by SEM-EDX. Correlation between the dentinal tubule penetration and bond strength.

Material	Dentinal Tubule Penetration (in µm)			POBS (in MPa)			Correlation
	Mean	SD	CV	Mean	SD	CV	
Ah Plus	60.57	20.57	0.34	3.36	2.07	0.61	0.401
FKG Hi-Flow	22.97	6.27	0.27	1.24	0.47	0.37	−0.898
Ah Bio	12.08	4.32	0.35	0.69	0.42	0.61	0.328

## Data Availability

The data presented in this study are available on request from the corresponding author.
